# *Gpnmb* and *Spp1* mark a conserved macrophage injury response masking fibrosis-specific programming in the lung

**DOI:** 10.1172/jci.insight.182700

**Published:** 2024-12-20

**Authors:** Emily M. King, Yifan Zhao, Camille M. Moore, Benjamin Steinhart, Kelsey C. Anderson, Brian Vestal, Peter K. Moore, Shannon A. McManus, Christopher M. Evans, Kara J. Mould, Elizabeth F. Redente, Alexandra L. McCubbrey, William J. Janssen

**Affiliations:** 1 Medical Scientist Training Program, University of Colorado School of Medicine, Aurora, Colorado, USA.; 2 Center for Genes, Environment, and Health, and; 3 Department of Medicine, National Jewish Health, Denver, Colorado, USA.; 4 Department of Medicine, University of Colorado School of Medicine, Aurora, Colorado, USA.; 5 Department of Pediatrics, National Jewish Health, Denver, Colorado, USA .

**Keywords:** Immunology, Inflammation, Fibrosis, Macrophages

## Abstract

Macrophages are required for healthy repair of the lungs following injury, but they are also implicated in driving dysregulated repair with fibrosis. How these 2 distinct outcomes of lung injury are mediated by different macrophage subsets is unknown. To assess this, single-cell RNA-Seq was performed on lung macrophages isolated from mice treated with LPS or bleomycin. Macrophages were categorized based on anatomic location (airspace versus interstitium), developmental origin (embryonic versus recruited monocyte derived), time after inflammatory challenge, and injury model. Analysis of the integrated dataset revealed that macrophage subset clustering was driven by macrophage origin and tissue compartment rather than injury model. *Gpnmb*-expressing recruited macrophages that were enriched for genes typically associated with fibrosis were present in both injury models. Analogous *GPNMB*-expressing macrophages were identified in datasets from both fibrotic and nonfibrotic lung disease in humans. We conclude that this subset represents a conserved response to tissue injury and is not sufficient to drive fibrosis. Beyond this conserved response, we identified that recruited macrophages failed to gain resident-like programming during fibrotic repair. Overall, fibrotic versus nonfibrotic tissue repair is dictated by dynamic shifts in macrophage subset programming and persistence of recruited macrophages.

## Abstract 2

Macrophages with fibrosis-associated programming exist in health, fibrosis, and non-fibrotic tissue repair. Their persistence and failed maturation into resident-like macrophages distinguishes fibrotic from non-fibrotic repair.

## Introduction

The acute respiratory distress syndrome (ARDS) is an inflammatory lung disease where the activities of immune cells contribute to alveolar and lung parenchymal tissue damage, leading to respiratory failure. Mortality in ARDS remains high at approximately 30%–40%, and effective therapies are lacking (1). Moreover, fibroproliferative remodeling of the lung occurs in up to 25% of survivors, resulting in pulmonary impairment that persists for months to years (2–4). The factors that drive the divergent outcomes of nonfibrotic repair versus fibroproliferative lung remodeling remain incompletely understood.

Macrophages play key roles in initiation and resolution of acute lung injury, and in lung fibrosis. Macrophages can promote tissue damage by producing proinflammatory cytokines that recruit and activate additional immune cells in the initial phase of lung injury. However, macrophages also contribute to tissue repair by clearing dead cells and debris and by producing proreparative cytokines and growth factors (5–7). Macrophages stimulate fibroblast proliferation and collagen synthesis via production of cytokines and growth factors, including CCL18, PDGF, FGF, and VEGF (8, 9). The macrophage activities that differentiate healthy repair from dysregulated repair with fibrosis are unclear. One possibility is that distinct subsets of macrophages mediate these different outcomes.

During health, the lungs are populated by 2 main subsets of tissue resident macrophages (10, 11). Resident airspace macrophages (RAM) occupy the airspace lumen where they contribute to lung homeostasis by recycling pulmonary surfactant, engulfing inhaled particulates and pathogens, and producing prorepair factors that maintain tissue integrity (12). In comparison, resident interstitial macrophages (RIM) exist in the interstitial compartment where they play roles in host defense, antigen presentation, and chemokine signaling. At least 2 subsets of RIM exist; however, their functions have not been completely elucidated (11, 13, 14). During inflammation, the macrophage pool expands as monocytes migrate to areas of injury where they mature into recruited airspace macrophages (RecAM) and recruited interstitial macrophages (RecIM).

The roles played by resident and recruited macrophage subsets in nonfibrotic injury models, such as the i.t. LPS model, have been previously characterized (12, 13, 15, 16). In these models, RAM programming remains relatively stable across time and is consistent with homeostatic functions. In comparison, RecAM augment inflammation during early time points but then transition to reparative programming as inflammation resolves (15, 17). RecIM express IFN response genes and monocyte marker genes and adopt RIM-like programming over time (13). Whether these populations have similar programming in fibrotic repair is unknown.

In mouse models of lung fibrosis, such as the bleomycin model, macrophages have been implicated as central drivers of the fibrotic response (18–21). While RAM programming remains fairly stable over time, RecAM programming is dynamic following bleomycin injury and includes enrichment for profibrotic genes. Importantly, strategies that block monocyte recruitment or lead to depletion of recruited lung macrophages attenuate fibrosis in fibrotic lung injury models (18, 19, 21, 22). While the aforementioned studies targeted IMs nonspecifically along with RecAM, little is known about the functions of RIM or RecIM in the bleomycin model.

Within the broader macrophage populations defined by origin and tissue compartment, transcriptomic analyses have described additional heterogeneity. Single-cell RNA-Seq (scRNA-Seq) studies in humans with ARDS have implicated a subset of macrophages with increased inflammatory programming associated with more severe disease (23, 24). Up to 35% of patients hospitalized with COVID-19 ARDS have had fibrotic changes on chest CT, implicating a role for profibrotic macrophage activity. However, we are unaware of any studies comparing scRNA-Seq data of lung macrophages from patients with COVID-19 with and without fibrosis (25–27). Studies of macrophages from idiopathic pulmonary fibrosis (IPF) identified a unique subset of AMs with profibrotic gene expression in IPF samples, and IPF AMs differed functionally from healthy control AMs (28–30). However, because these studies were performed in isolation and with single time points, the relationship of repair- and fibrosis-associated subsets to each other remains unknown. These knowledge gaps have limited our ability to identify outcome-associated biomarkers or develop targeted therapeutics.

To determine differences in macrophage subsets in nonfibrotic repair versus dysregulated repair with fibrosis, we employed 2 well-described yet distinct mouse models that recapitulate important features of human ARDS. To model efficient, nonfibrotic repair we used i.t. instillation of *E*. *coli* LPS. LPS activates TLR4 innate immune signaling, resulting in neutrophil and macrophage accumulation in the lungs, disruption of the alveolar-endothelial barrier, and pulmonary edema, which replicate features of human ARDS (31–33). LPS injury is self-limited and spontaneously resolves in less than 2 weeks with near-complete restoration of normal lung tissue architecture (34). To model dysregulated lung repair with fibrosis, we used single-dose i.t. bleomycin. Bleomycin induces oxygen radical-mediated DNA damage and cell death and recapitulates the early neutrophil accumulation (like LPS), but it is followed by an aberrant tissue repair response as seen in patients with fibrotic ARDS (35–37).

The goal of this study was to identify macrophage subsets or features unique to fibrosis versus nonfibrotic repair. To accomplish this, we compared single-cell transcriptomics of pulmonary macrophages from resolving (LPS) and fibrotic (bleomycin) injury models over time. We hypothesized that contrasting these 2 models over the time course of disease would identify a unique subset of profibrotic macrophages present in the bleomycin model and a subset of prorepair macrophages unique to the LPS model. Comparison of these groups would allow us to understand how macrophages facilitate nonfibrotic repair versus fibrosis.

This study is, to our knowledge, the first to directly compare macrophages from 2 distinct injury models representing nonfibrotic repair and dysregulated repair with fibrosis at multiple time points after injury initiation. We leveraged lineage tracing and tissue compartment–specific labeling to demonstrate that macrophage tissue location and origin, rather than injury model or time, are the major drivers of distinct macrophage transcriptional profiles. Furthermore, we demonstrate the absence of a profibrotic macrophage subset unique to the bleomycin model and instead conclude that recruited macrophages with “fibrotic” genes are a conserved feature of injury. Shifts in macrophage programming over time and the persistence of recruited macrophages are associated with prolonged inflammation and fibrosis.

## Results

### scRNA-Seq of lung macrophages over time courses of LPS- and bleomycin-induced lung injury and repair.

The goal of this study was to identify differences in macrophage subsets between self-limited lung injury with nonfibrotic repair and prolonged injury with dysregulated repair and fibrosis. To achieve this, we employed 2 mouse models to represent these divergent outcomes of ARDS. LPS induces acute lung injury that fully resolves without fibrosis within 2 weeks, while bleomycin induces injury that progresses to fibrosis before eventual resolution over 2 months (Supplemental Figure 1; supplemental material available online with this article; https://doi.org/10.1172/jci.insight.182700DS1).

**Figure 1:**
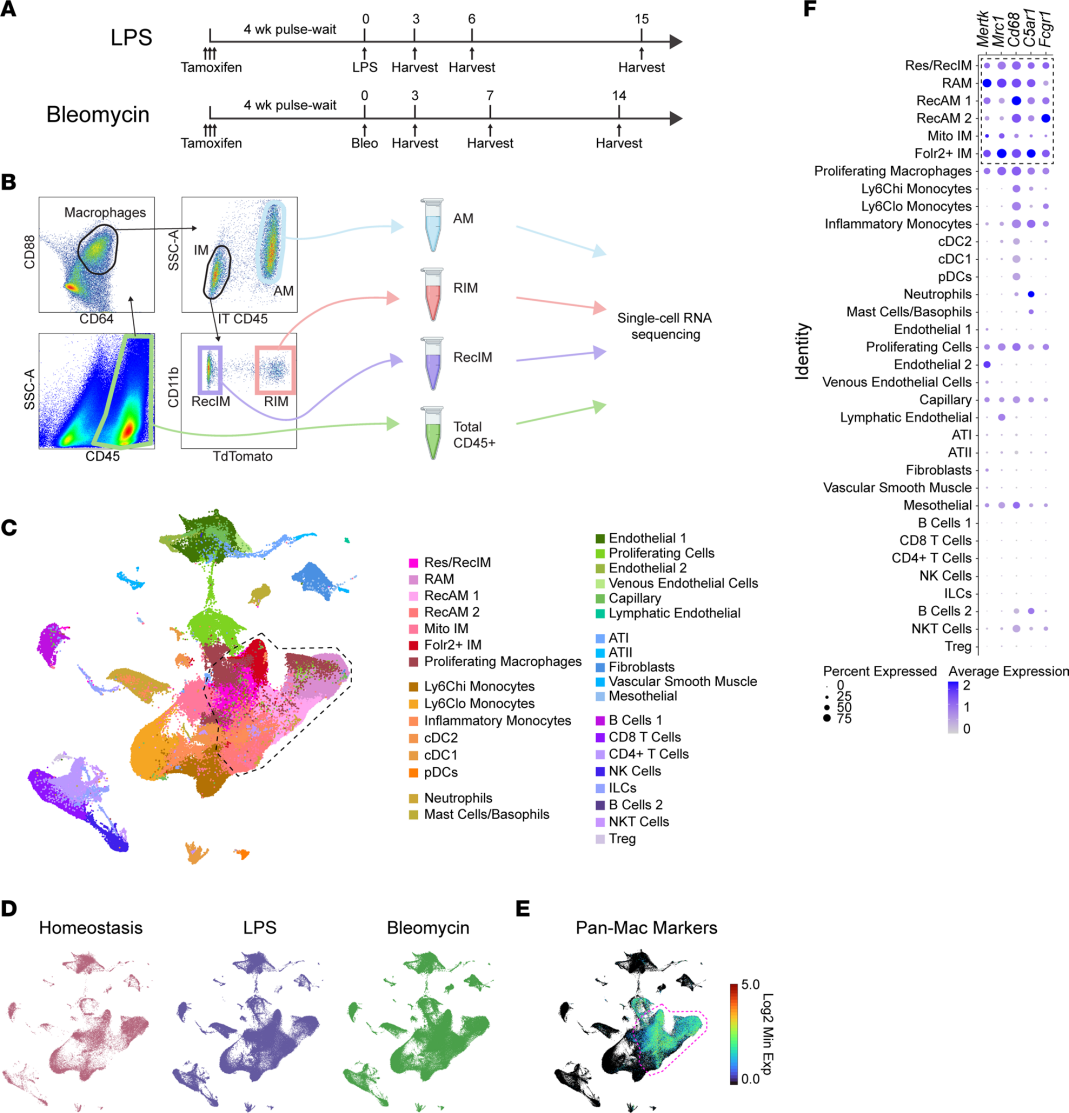
Macrophage isolation and scRNA-Seq following bleomycin and LPS-induced lung injury. (**A**) Timeline of interventions in experimental models. Tamoxifen was given to label resident interstitial macrophages followed by a 4-week wait period to allow clearance of labeled monocytes from the circulation. Mice were treated with i.t. LPS (20 μg) or bleomycin (1.5 U/kg) and were euthanized at the times indicated. Anti-CD45 antibody was instilled into the lungs immediately after euthanasia. (**B**) Four groups of cells were isolated from digested lung tissues using FACS. These included airspace macrophages (AMs), resident IMs (RIM), recruited IMs (RecIM), and general leukocytes (CD45^+^). AMs versus IMs were distinguished based on i.t. administered anti-CD45 labeling. Resident versus recruited IMs were distinguished by tdTomato expression. (**C**) Fully integrated UMAP including all sorted cells from day 0; LPS days 3, 6, and 15; and bleomycin days 3, 7, and 14. Clusters were manually annotated based on cluster-defining genes. Macrophage clusters are outlined in the dashed black line. (**D**) UMAP split to show homeostasis, LPS, and bleomycin samples. (**E**) Feature plot of the log_2_ minimum expression of mouse panmacrophage markers *Fcgr1*, *C5ar1*, *CD68*, *Mrc1*, and *Mertk*. (**F**) Average expression and percent of cells expressing panmacrophage marker genes in each cluster. Macrophage clusters in the dashed black box were used in subsequent analyses.

A long-standing barrier in the field is a lack of cell surface markers that reliably distinguish resident from recruited IMs over the course of inflammation. To address this challenge, we pulsed Cx3cr1-TdTomato mice with tamoxifen to label all RIM. Since this strategy also labeled circulating monocytes, we waited for 4 weeks to allow monocytes to turn over and then administered LPS or bleomycin (38). Lungs were harvested on days 3, 6, and 15 after LPS and days 3, 7, and 14 after bleomycin (Figure 1A). These time points correspond to early neutrophilic inflammation, early repair, and late repair in both models (Supplemental Figure 1). Immediately after euthanasia, anti–mouse CD45 antibody was instilled i.t. (IT CD45) to label all immune cells in the airspaces (13). Lungs were digested, and 3 macrophage populations were simultaneously sorted: all AMs, RecIM, and RIM (Figure 1B and Supplemental Figure 2). While this sort strategy enabled lineage tracing and anatomic localization, it masked information regarding relative population sizes between sorts. To address this, we simultaneously collected a fourth set of sorted cells from each lung containing total CD45^+^ leukocytes. Each population was sequenced separately to preserve spatial and lineage identification.

Individual sequencing libraries were integrated into a single dataset incorporating all time points and models. From this common dataset, Seurat clustering yielded 34 distinct cell types that were manually annotated using LungMAP (39) murine marker genes (Figure 1C and Supplemental Figure 3). As anticipated, the dataset was greatly enriched for macrophage populations. Other myeloid and lymphoid cell subsets were also captured in the CD45^+^ sort libraries as well as small numbers of structural tissue cells, particularly endothelial cells. Splitting the Uniform Manifold Approximation and Projection (UMAP) by homeostasis versus injury models revealed that lung structural cells and tissue resident immune cells dominate at homeostasis. In lungs from LPS- and bleomycin-treated mice, additional macrophage clusters were present and other leukocyte clusters were expanded, consistent with inflammatory responses (Figure 1D). Pan-macrophage markers *Fcgr1*, *C5ar1*, *Cd68*, *Mertk*, and *Mrc1* were used to identify macrophage clusters in the aggregate dataset (Figure 1, E and F). The cluster annotated as “Mito IM” was excluded from further analysis due to high enrichment for mitochondrial RNA transcripts.

### Macrophage clusters are defined by origin and compartment.

**Figure 2:**
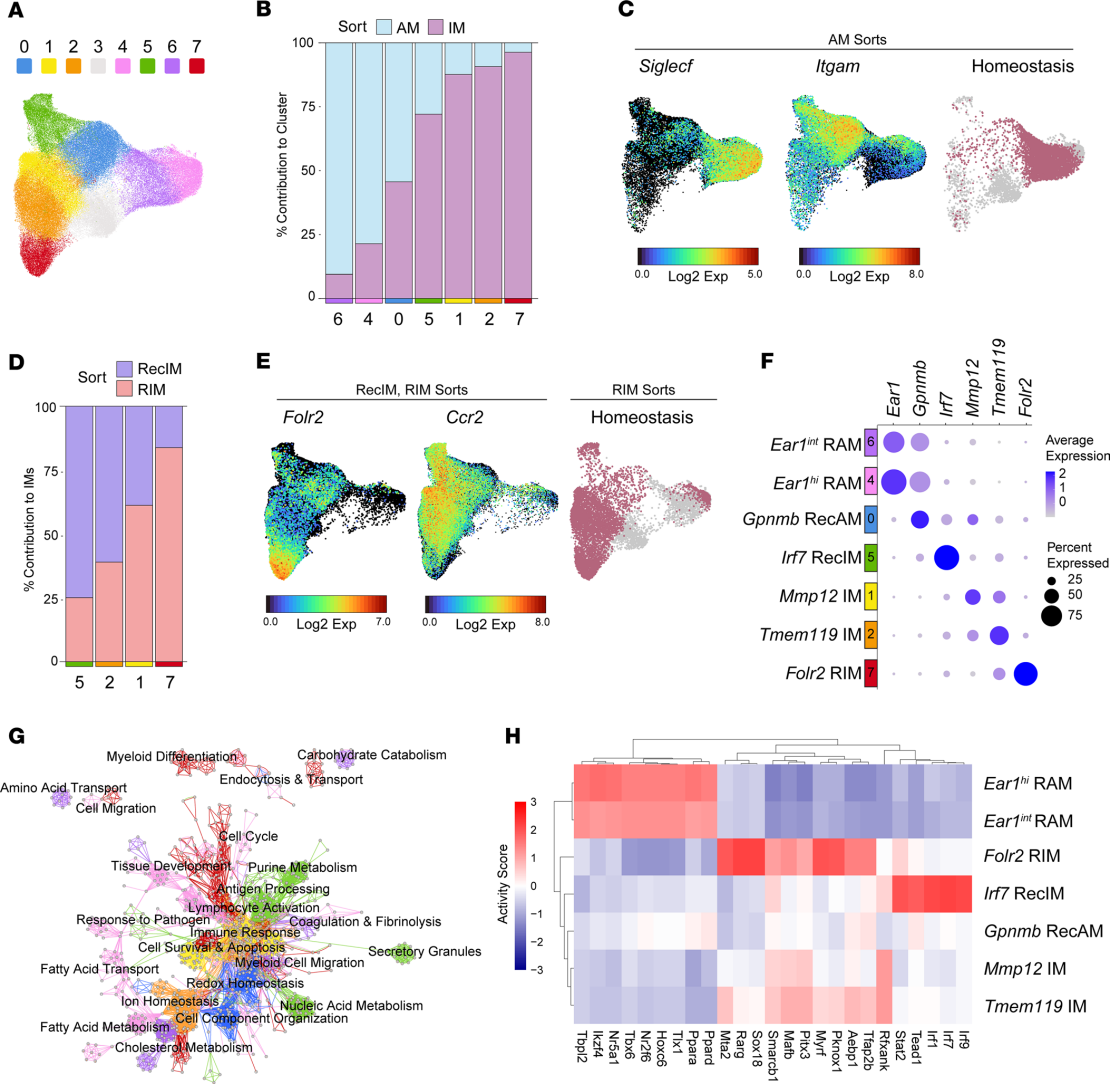
Macrophage clusters correspond to compartment and origin. (**A**) Macrophage-specific UMAP generated by reclustering macrophage clusters from the global UMAP. (**B**) Relative contribution of alveolar macrophages (AM) versus interstitial macrophages (IM) to each cluster, based on sort (AM versus IM) from which the cells were derived. (**C**) Feature plots of the AM sort from all time points and models showing expression of *Siglecf* and *Itgam*, and a UMAP showing the AM sorts exclusively from homeostasis samples. (**D**) Fraction of cells derived from RIM versus RecIM sorts for IM clusters. (**E**) Feature plots of IMs from all time points and models showing expression of *Folr2* and *Ccr2*, and a UMAP showing IMs derived exclusively from homeostasis samples. (**F**) Dot plot of selected marker gene expression for the 7 macrophage clusters. (**G**) Cytoscape network visualization of GO pathways enriched in the marker lists for each cluster. Nodes correspond to individual pathways, and edges connect nodes with shared genes. Edge color indicates the cluster that is most highly enriched for that pathway; however, multiple clusters may be enriched for a given pathway. (**H**) DecoupleR transcription factor inference heatmap showing activity scores of the top 25 most variable transcription factors between the 7 clusters.

To enable granular analysis of macrophage subsets, clusters identified as macrophages in the global UMAP were subclustered, identifying 8 macrophage subsets (Figure 2A). Cluster 3 was removed from subsequent analysis because it lacked differentially expressed genes (DEGs) and was instead defined by overall low gene expression relative to other clusters. Proliferating cells were not included in the subclustering.

Because pulmonary macrophage programming is influenced by location, we first sought to assign each of the remaining 7 clusters to the airspace or interstitial compartment. Using the sort information encoded in the dataset, the proportion of cells derived from alveolar macrophage (AM) versus interstitial macrophage (IM) sorts was determined for each cluster (Figure 2B). Clusters 4 and 6 were predominately AMs. Cluster 0 was split between AMs and IMs, while clusters 1, 2, 5, and 7 were dominated by IMs.

Since macrophage programming is tightly linked to ontogeny during inflammation, we next sought to identify the origin of cells within each cluster (12). In the airspace, RAM and RecAM can be reliably distinguished by expression of Siglec-F (*Siglecf*) and CD11b (*Itgam*) (40). Accordingly, we found clusters 4 and 6 had high expression of *Siglecf*, suggesting that these clusters were composed of RAM. In comparison, cluster 0 exhibited high *Itgam* expression consistent with RecAM. To further confirm origin, we mapped cells in AM sorts from homeostasis. Homeostatic AMs overlaid clusters 4 and 6, confirming their RAM identity, while cluster 0 was nearly absent at homeostasis (Figure 2C). To identify the origin of IM clusters, we determined the proportion of resident versus recruited cells within each IM cluster using RIM and RecIM sort information. This suggested that clusters 1 and 2 were a mix of RIM and RecIM, cluster 5 was predominately composed of RecIM, and cluster 7 contained RIM (Figure 2D). To confirm these findings, we evaluated IM clusters for expression of selected genes. Since no markers exist that reliably distinguish all RIM subsets from RecIM subsets, we evaluated *Folr2*, which marks a subset of RIM, and *Ccr2*, which marks all RecIM but is also expressed by a subset of RIM (11, 13). We again used the homeostasis samples to confirm that cluster 7 is composed of RIM, cluster 5 contains RecIM, and clusters 1 and 2 have both RIM and RecIM (Figure 2E).

After identifying the contribution of compartment and origin to cell clustering, we sought to define specific marker genes for each cluster. We first identified cluster marker genes within each sample, and we next determined a conserved set of markers for each cluster that were significantly differentially expressed in at least 80% of samples. This led to identification of specific, conserved marker genes for each cluster except for clusters 4 and 6, which had overlapping markers, albeit with different expression levels (Supplemental Table 1). As a final step, we combined assessments of maker genes, compartment, and origin to assign the following identifiers to each cluster: 0 = *Gpnmb* RecAM, 1 = *Mmp12* IM, 2 = *Tmem119* IM, 4 = *Ear1^hi^* RAM, 5 = *Irf7* RecIM, 6 = *Ear1^int^* RAM, and 7 = *Folr2* RIM (Figure 2F). These cluster identities are similar to macrophage subsets described in previous studies (11, 13).

Using the conserved markers for each cluster, we performed gene set enrichment analysis to infer cluster-specific functions. In the Cytoscape Enrichment Map network visualization tool, individual nodes correspond to Gene Ontology (GO) pathways and edges connect nodes with overlapping gene sets (41, 42). Edges were colored according to the cluster with the lowest adjusted *P* value, although multiple clusters may have significant enrichment for a given pathway (Figure 2G and Supplemental Table 2). As expected, RAM cluster marker genes were enriched in fatty acid, lipid, and cholesterol metabolism pathways, highlighting their roles in lung surfactant processing. Other pathways enriched in RAM related to endocytosis and tissue development, again suggesting homeostatic functions. In comparison, *Gpnmb* RecAM were uniquely enriched for pathways related to redox stress, immune activation, and cellular reorganization. In the IM compartment, *Folr2* RIM were enriched for pathways related to lung morphogenesis, blood vessel development, and myeloid chemotaxis and immune activation, suggesting that these cells play key homeostatic roles by maintaining lung structure and immune cell tone. *Mmp12* IM were enriched for antigen processing and presentation pathways but were also enriched for cellular response pathways containing *Egr1*, a transcription factor shown to suppress the inflammatory response (43). *Tmem119* IM shared enrichment for inflammatory response pathways with *Mmp12* IM but were uniquely enriched for complement activation and creatine metabolism pathways. Lastly, *Irf7* RecIM were enriched for immune-related pathways, including response to pathogens, secretory granules, and antigen processing and lymphocyte activation, suggesting a proinflammatory function.

To identify putative transcription factors that underlie distinct subset identities, we performed transcription factor inference analysis via decoupleR and calculated the top 25 most variable transcription factors between clusters (Figure 2H) (44). *Ear1^hi^* and *Ear1^int^* RAM shared 9 transcription factors and were characterized by PPAR transcription factors, which are key regulators of lipid and carbohydrate metabolism (45, 46). A key transcription factor of the *Folr2* RIM was *Mafb*, which is known to drive antiinflammatory programming in macrophages (47). *Mafb* activity was also higher in *Mmp12* IM and *Tmem119* IM and may be a driver of monocyte-to-IM maturation (48). *Irf7* RecIM had high activity of proinflammatory transcription factors downstream of type I and type III IFN signaling. Lastly, the *Gpnmb* RecAM had no unique high-activity transcription factors, which may reflect the low number of conserved markers (Supplemental Table 1).

### Macrophage cluster sizes vary by time and injury model.

**Figure 3:**
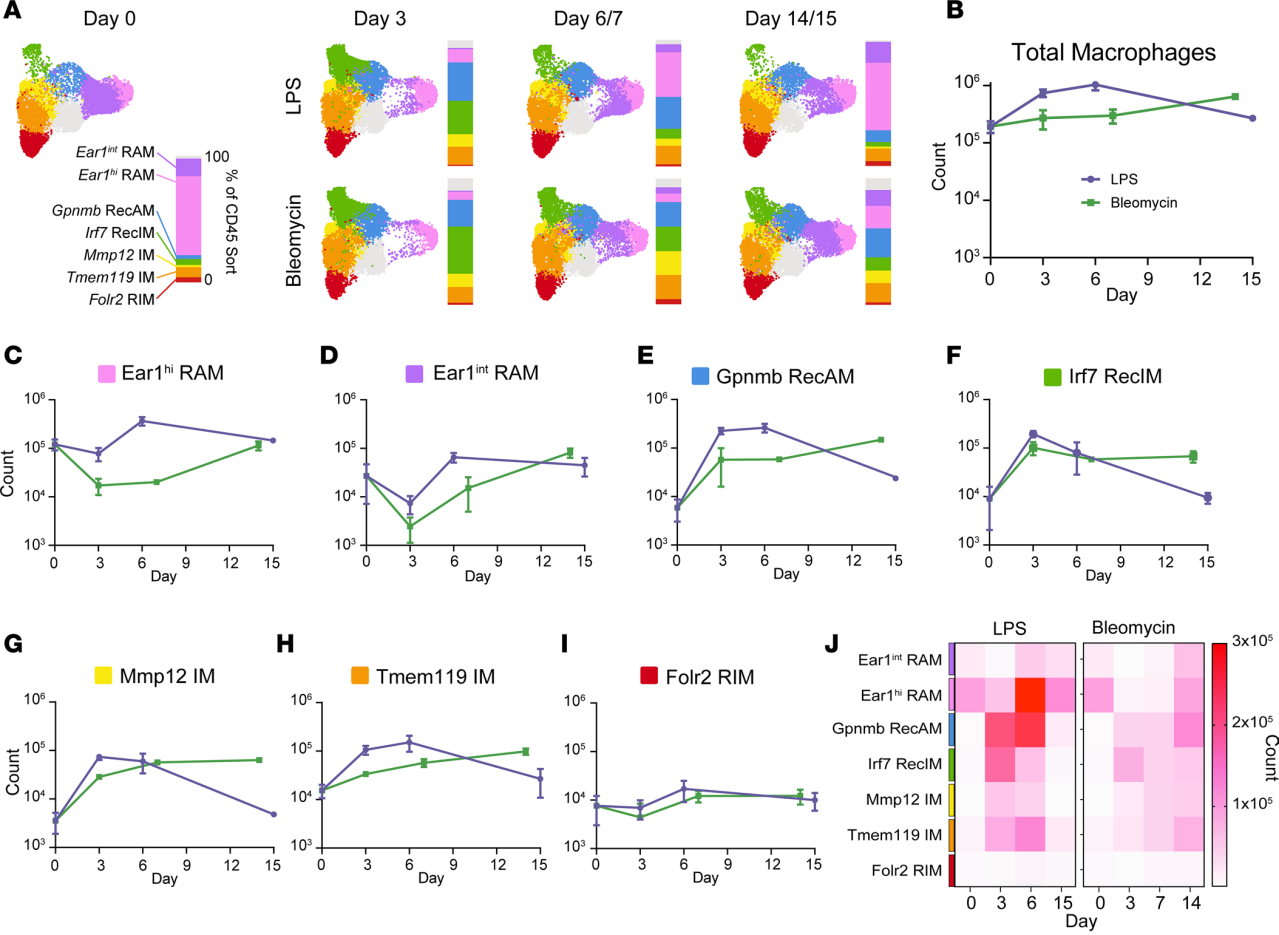
Macrophage cluster sizes vary over time and between models. (**A**) Macrophage UMAP split by model and time point. Vertical bars show the percent contribution of each cluster in the CD45^+^ sort to the macrophage pool for each model and time point. (**B**) Total macrophage numbers in the lungs from mice treated with i.t. LPS or bleomycin. (**C**–**I**) Estimated cell numbers for clusters in LPS (purple) versus bleomycin (green). (**J**) Heatmap of cell counts for each cluster at each model time point. Line graphs in **B**–**I** show mean ± SEM.

Having determined the compartment and origin of each macrophage cluster, we next asked whether any clusters were unique to an injury model or time point. To eliminate proportion bias from macrophage-enrichment sorts, macrophage cluster proportions were calculated from CD45^+^ sorts (Figure 3A). All clusters were present at all postinjury time points in both models, indicating that Seurat clustering did not identify exclusively model- or time-specific clusters. Principal component analysis of average log-normalized expression of cells from AM, RIM, and RecIM sorts at each time point in each model confirmed that compartment and origin were the strongest determinants of clustering, followed by time point and injury model (Supplemental Figure 4).

Although none of the clusters were model specific or time specific, we observed substantial differences in cluster proportions between models and time points (Figure 3A). Since total macrophage numbers also differ between the LPS and bleomycin models over time (Figure 3B), we combined them with cluster proportions from CD45^+^ sorts (Figure 3A) to determine total macrophage numbers for each cluster over time. This revealed model-specific differences in population kinetics of resident and recruited macrophage clusters. In particular, *Ear1^hi^* and *Ear1^int^* RAM numbers rapidly decreased at early time points following bleomycin challenge before returning to baseline at day 14, whereas in the LPS model, they remained stable at early time points and then transiently expanded at day 6 before eventually returning to baseline (Figure 3, C and D). In contrast, RecAM and RecIM clusters exhibited rapid expansion at early time points in both models. However, in LPS-treated mice RecAM and RecIM clusters returned to baseline by day 15, whereas in bleomycin-treated mice, they remained elevated (Figure 3, E–G). *Folr2* RIM numbers remained stable throughout inflammation in both models (Figure 3I). In summary, no model- or time-specific macrophage clusters were identified. Instead, model-specific differences were found by examining cluster sizes over time, with early, transient loss of RAM and later persistence of RecAM and RecIM in the bleomycin model compared with LPS (Figure 3J). Notably, numbers of recruited macrophages were remarkably similar between models during the initiation of inflammation.

### Macrophages with profibrotic gene expression are found in lungs from bleomycin- and LPS-treated mice and in humans with fibrotic and nonfibrotic diseases.

**Figure 4:**
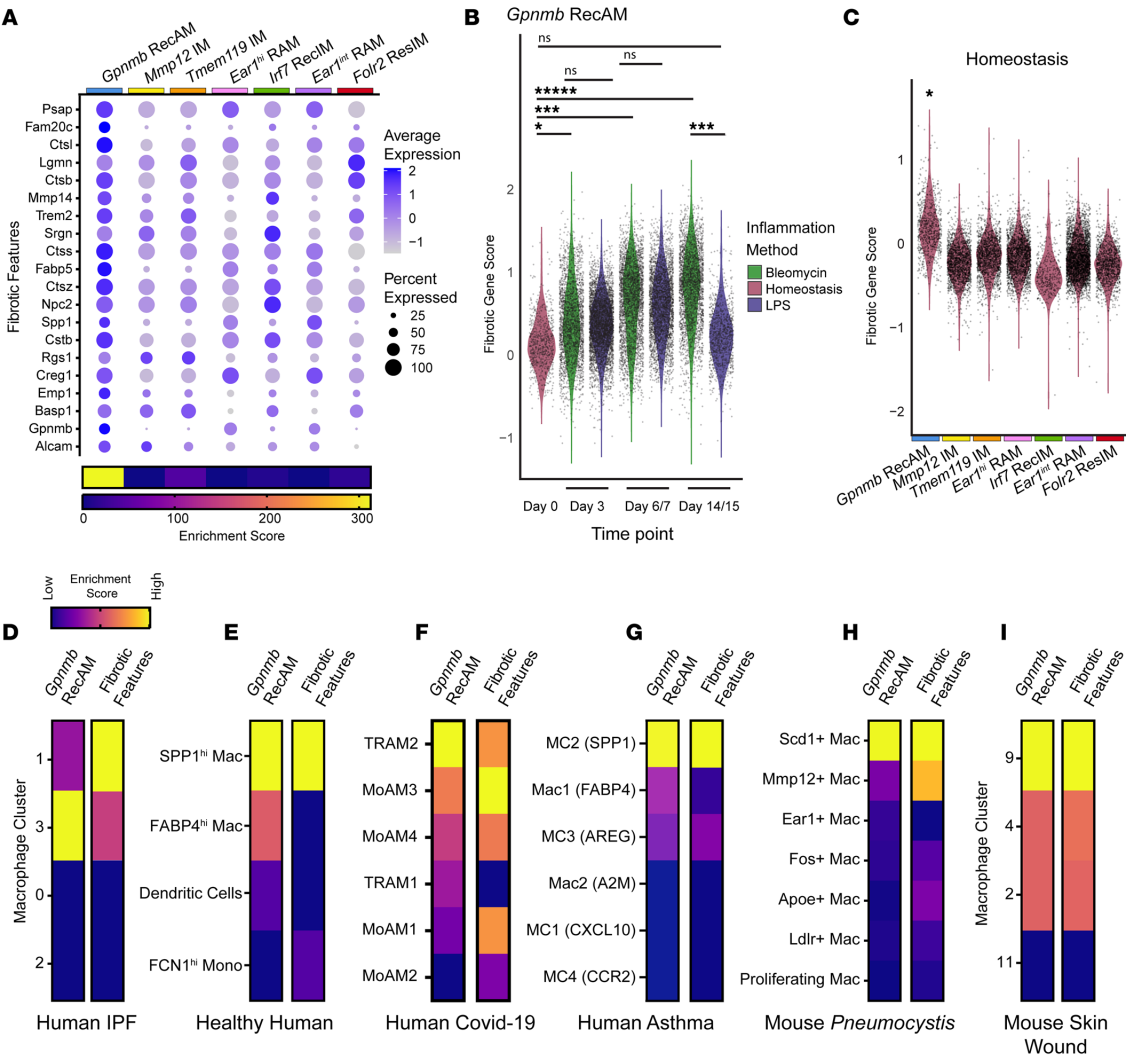
*Gpnmb* RecAM macrophages are found in fibrotic and nonfibrotic lung disease in mice and humans. (**A**) Dot plot of expression of fibrosis-associated macrophage marker genes (fibrotic features) in macrophage clusters, scaled across rows. For each cluster, enrichment scores were calculated comparing the overlap of conserved cluster markers and fibrotic features to the expected number of overlapping genes by chance. These are represented in the heatmap. Data are from LPS and bleomycin models and include all time points. (**B**) Violin plot of fibrotic features gene set scores in *Gpnmb* RecAM from each model and time point. (**C**) Violin plot of fibrotic features gene set scores in macrophage clusters from homeostasis. (**D**–**G**) Heatmap of enrichment scores of the *Gpnmb* RecAM gene set and the fibrotic features gene set in macrophage subset marker lists from: human IPF lungs (**D**), healthy human bronchoalveolar lavage (**E**), human severe COVID-19 (**F**), allergic asthmatics and nonasthmatic controls (**G**), nonfibrotic murine models of pulmonary pneumocystis infection (**H**), and skin wounds in healthy and diabetic mice (**I**). Hypergeometric tests in **B** and **C**. *P < 0.05, ***P < 0.0005, *****P < 0.000005.

We initially hypothesized that our analysis would reveal a fibrosis-associated macrophage subset unique to the bleomycin model; however, we found no model-specific clusters. We therefore investigated the expression of fibrosis-associated genes in our macrophage clusters using a published list of fibrosis-associated macrophage genes validated in lung tissue from humans with pulmonary fibrosis and mice with asbestos- and bleomycin-induced fibrosis (20). We compared expression of these “fibrotic features” between the 7 macrophage clusters without separating cells from models or time points (Figure 4A). All clusters expressed genes from the list; however, comparison of the conserved cluster marker lists to the fibrotic features list demonstrated that *Gpnmb* RecAM were highly enriched for these fibrotic features.

To determine if the expression of these fibrotic features in *Gpnmb* RecAM was model or timespecific, we calculated a gene score to compare the average expression of fibrotic features between *Gpnmb* RecAM from each time point in each model. Briefly, fibrotic features scores were calculated for each cell in the *Gpnmb* RecAM cluster by taking the average of the centered and scaled expression of genes in the fibrotic features gene set. Fibrotic feature scores were elevated in *Gpnmb* RecAM from all LPS and bleomycin inflammatory time points compared with homeostasis, except for LPS day 15 (Figure 4B). Moreover, Fibrotic Feature scores were not different between the corresponding LPS and bleomycin time points except for day 14 versus 15 (day 14/15). Notably, comparison of fibrotic feature scores for all macrophage subsets at homeostasis continued to identify *Gpnmb* RecAM as “fibrotic” relative to other macrophage clusters (Figure 4C). Taken as a whole, these data show that *Gpnmb* RecAM had greater expression of fibrosis-associated genes than other macrophage subsets at homeostasis. Moreover, the expression of these fibrosis-associated genes increased in *Gpnmb* RecAM in both the LPS and bleomycin models in parallel, only diverging at day 14/15.

Interestingly, analysis of the fibrotic feature score at each time point in each model for the remaining macrophage clusters revealed a similar pattern to that observed in *Gpnmb* RecAM (Supplemental Figure 5). While fibrotic feature scores were much lower in the other macrophage clusters overall compared with *Gpnmb* RecAM, within each cluster, the scores were similar between LPS and bleomycin at days 3 and 6/7 and only diverged at day 14/15 for most clusters. This suggests that expression of genes in the fibrotic features list is not unique to fibrosis but rather represents a global response of macrophages to tissue injury or inflammation.

Given these findings, we hypothesized that homologs to the murine *Gpnmb* RecAM subset would be present in humans with both fibrotic and nonfibrotic lung disease. To simultaneously test this hypothesis and validate that *Gpnmb* RecAM corresponded to “profibrotic” macrophages described by others, we generated a *Gpnmb* RecAM gene signature using cluster-specific conserved markers (Supplemental Table 1). We next tested markers of macrophage subsets previously identified in human IPF lungs for enrichment of both the *Gpnmb* RecAM gene signature and the fibrotic features signature (49, 50). Two AM clusters from the human IPF dataset were enriched for both the *Gpnmb* RecAM gene signature and the fibrotic features signature (Figure 4D). The authors identified cluster 1 in this dataset as profibrotic (29). This suggests that *Gpnmb* RecAM share common markers with so-called profibrotic macrophages and that a *Gpnmb* RecAM-like macrophage subset is present in human IPF.

Although recruited macrophage numbers are low in health, a small population of macrophages expressing profibrotic genes such as *SPP1* has been described in BAL from humans (51, 52). Because *Gpnmb* RecAM had higher expression of the fibrotic features relative to other macrophages at homeostasis, we hypothesized that *Gpnmb* RecAM corresponded to a BAL macrophage subset from healthy humans. Indeed, enrichment analysis revealed that genes that define the *SPP1^hi^* macrophage cluster from healthy humans were enriched for both fibrotic feature genes and *Gpnmb* RecAM genes (Figure 4E) (52). Thus, macrophages with these gene signatures exist in small numbers at baseline in humans and in mice. This demonstrates a clear limitation in the utility of “fibrotic feature” genes to indicate fibrosis. The presence of a macrophage subset enriched for “profibrotic” genes during health further suggests that this subset is not unique to fibrosis nor is it sufficient to cause fibrosis. Because *Gpnmb* RecAM gene expression overlaps with “fibrotic” cluster gene signatures but not fibrotic repair outcomes, these signatures may instead identify a reparative subset involved in both tissue homeostasis and a general response to injury.

To test this hypothesis, we assessed whether *Gpnmb* RecAM-like macrophages could be found in other nonfibrotic human diseases and mouse models. We calculated enrichment scores for both the *Gpnmb* RecAM gene signature and the fibrotic features gene signature in marker lists from published scRNA-Seq datasets from 2 distinct forms of human lung disease (COVID-19 and asthma), a mouse model of live *Pneumocystis* lung infection, and mouse macrophages isolated from skin wounds of healthy and diabetic mice (Supplemental Table 3) (24, 29, 52–54). The *Gpnmb* RecAM gene signature had significant enrichment in monocyte-derived macrophages (MoAM3) and SARS-CoV-2–infected RAM (TRAM2) in BAL from patients with severe COVID-19 (Figure 4F). Since severe COVID-19 can result in pulmonary fibrosis, we sought to compare macrophages from a disease that is not associated with interstitial fibrosis (25, 26, 54). We therefore compared macrophages from bronchial brushings of patients with allergies and asthma against patients without allergies who have asthma (55). *Gpnmb* RecAM genes were enriched in the markers for an *SPP1*-expressing monocyte-macrophage subset, as were the fibrotic features genes (Figure 4G). Similarly, *Gpnmb* RecAM and fibrotic features gene signatures were significantly enriched in macrophage subsets from mice with nonfibrotic lung injury from *Pneumocystis jirovecii* (Figure 4H) and sterile skin wounds (Figure 4I). Collectively, these data suggest a paradigm where the core biology regulating macrophage recruitment and maturation in response to injury is conserved across species and tissues. The most easily identifiable signature genes, including *Spp1* and *Gpnmb*, mark a macrophage subset with a shared injury response identity that has been mischaracterized as uniquely or innately profibrotic.

### Gpnmb RecAM transcriptional profiles differ between LPS and bleomycin.

**Figure 5:**
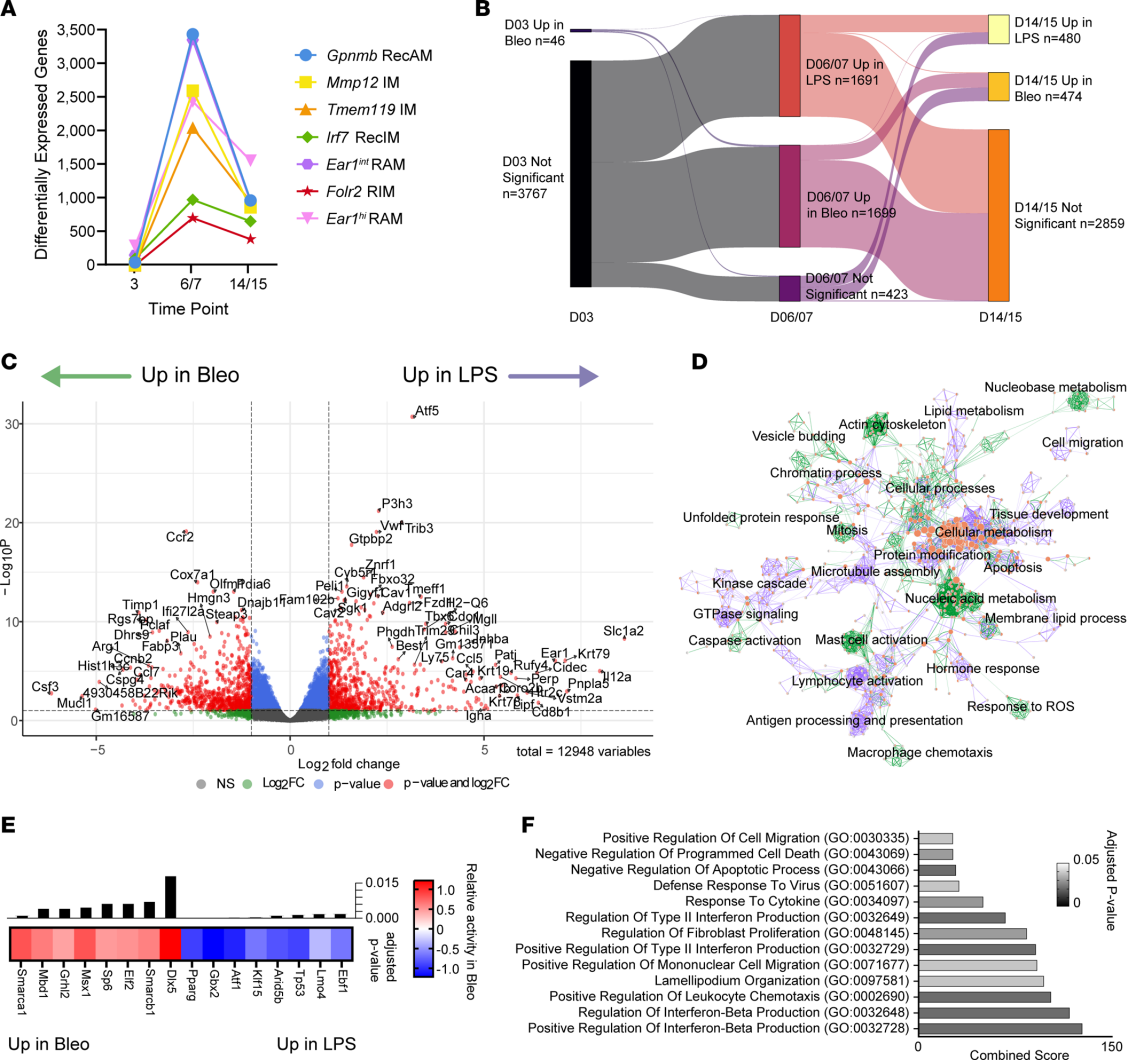
Differential gene expression in *Gpnmb* RecAM from LPS versus bleomycin. (**A**) Number of DEGs between LPS and bleomycin within each cluster at corresponding time points (FDR < 0.05). (**B**) Sankey plot of DEGs from *Gpnmb* RecAM at each comparison time point. Significance is based on logFC > 0.5 and FDR < 0.05. (**C**) Volcano plot of differentially expressed genes (DEGs) for *Gpnmb* RecAM LPS day 6 versus bleomycin day 7. (**D**) Cytoscape network visualization of *Gpnmb* RecAM pathway analysis performed on DEGS up in bleomycin day 7 versus up in LPS day 6. Circles represent enriched pathways and are clustered by similarity. Lines connecting the circles represent genes that overlap between pathways. Circle halves are shaded from grey (no pathway enrichment) to red (high enrichment) with left sides representing LPS and right sides representing bleomycin. Line colors mark the primary group showing significant pathway enrichment, either bleomycin (green) or LPS (purple). (**E**) Heatmap of transcription factor activity inference scores that were significantly different between *Gpnmb* RecAM from bleomycin day 7 versus LPS day 6. Only the top 8 transcription factors with the lowest adjusted *P* values for each injury model are shown. (**F**) Top 10 GO pathways enriched in the 230 DEGs upregulated in *Gpnmb* RecAM from bleomycin at both day 7 and day 14 compared with LPS day 6 and 15. Adjusted *P* < 0.05.

Since the presence of *Gpnmb* RecAM is not sufficient to predict repair outcome, we hypothesized that the conserved injury response signature driving *Gpnmb* RecAM clustering might hide subtler model-specific transcriptional differences important to fibrosis. To uncover transcriptional changes that might contribute to fibrotic versus nonfibrotic repair outcomes, we tested for differences in macrophage gene expression between LPS and bleomycin models at corresponding time points within each cluster. Numbers of DEGs between LPS- versus bleomycin-derived macrophages were relatively low for all clusters at day 3 (Figure 5A). However, the number of DEGs increased substantially for most clusters at day 6/7, particularly *Gpnmb* RecAM and *Ear1^int^* RAM. These data suggest that much of the initial macrophage injury response is conserved between models before diverging at later time points.

Because *Gpnmb* RecAM demonstrated enrichment for fibrosis-associated genes (Figure 4, A–C), we interrogated the LPS versus bleomycin DEG list for genes from the fibrotic features list. Of the 20 genes in the fibrotic features list, only *Spp1* and *Emp1* were in the top 100 DEGs increased in bleomycin relative to LPS at every time point. Six of 20 were significantly increased in bleomycin at day 6/7, and 12 of 20 were increased at day 14/15 compared with LPS (Supplemental Table 4).

We next sought to better understand *Gpnmb* RecAM DEGs across time. Overall, only 10 genes were upregulated in bleomycin at all 3 time points, and most of the DEGs at day 6/7 did not remain differentially expressed at day 14/15 (Figure 5B). Since DEG numbers were greatest in *Gpnmb* RecAM at days 6/7, we next focused on this time point. *Gpnmb* RecAM from bleomycin-treated mice had greater expression of chemokines associated with fibrosis, including *Ccl7*, *Ccl17*, *Ccl2*, and *Ccl9* (56). In comparison, *Gpnmb* RecAM from LPS-treated mice had higher expression of genes associated with RAM identity, including *Ear1*, *Car4*, and *Plet1* (Figure 5C and Supplemental Table 5).

To determine the differences in putative functions of *Gpnmb* RecAM from LPS- versus bleomycin-treated mice at days 6/7, we performed pathway analysis using DEGs that were increased in each model (Figure 5D and Supplemental Table 6). Here we found that LPS-derived cells were enriched for phospholipid and lipid metabolism, suggesting a role for the GM-CSF/PPARγ axis and perhaps ongoing conversion to a homeostatic RAM-like state. LPS-derived *Gpnmb* RecAM were also enriched for pathways related to antigen processing and tissue development, which are also indicative of homeostatic functions. By contrast, *Gpnmb* RecAM from bleomycin-treated mice were enriched for numerous inflammation and stress-related pathways such as mast cell activation, unfolded protein response, and response to ROS. While we previously removed actively proliferating cell clusters from the scRNA-Seq dataset, we found enrichment for cell cycle and mitosis pathways suggesting recent proliferation. This suggests that proliferation may contribute to continued expansion of the *Gpnmb* RecAM pool in bleomycin.

To determine putative transcription factors responsible for differences in gene expression, we performed transcription factor activity inference analysis for *Gpnmb* RecAM from LPS day 6 versus bleomycin day 7 (Figure 5E). LPS-derived *Gpnmb* RecAM were found to have relatively higher activation of transcription factors associated with lipid homeostasis and negative regulation of inflammation, including *Pparg*, *Klf15*, and *Arid5b*. *Pparg* is widely recognized as a key transcription factor that regulates RAM identity and lipid homeostasis, whereas *Klf15* and *Arid5b* have been implicated in suppressing inflammation (57, 58). Transcription factors with increased predicted activity in *Gpnmb* RecAM from bleomycin relative to LPS included *Hoxa11*, *Msx1*, and *Dlx5* (Supplemental Table 7). *Hoxa11* and *Msx1* have been shown to increase profibrotic factors such as PDGF and TGF-β (59, 60). *Dlx5* has been found to bind to and suppress PPARγ activity in mesenchymal stem cells, which, if it occurs in RecAM, could mediate inhibition of RAM-like programming (61, 62).

These differences suggest that day 6/7 may represent a point of divergence in nonfibrotic versus fibrotic repair. As a final step, we identified genes that were consistently upregulated in bleomycin-derived *Gpnmb* RecAM at both days 7 and 14 relative to LPS days 6 and 15, hypothesizing that these might reflect gene signatures involved in the transition to fibrosis and related to the transcription factors from day 6/7. Of the 1,699 DEGs upregulated in bleomycin-derived *Gpnmb* RecAM at day 6/7, only 230 genes were also upregulated at day 14/15 (Supplemental Table 8). GO pathway analysis demonstrated enrichment for IFN production, chemotaxis, and cell survival (Figure 5F and Supplemental Table 8).

Taken as a whole, these analyses suggest that an additional, subtler layer of programming exists beneath the shared injury response that drives *Gpnmb* RecAM identity. Notably, examination of the 20 fibrotic feature genes showed that only *Spp1* and *Emp1* identified model-specific differences between *Gpnmb* RecAM at every time point, again highlighting that these genes mark a conserved injury response but largely fail to recognize biology correlated with fibrotic outcome. Bleomycin-derived *Gpnmb* RecAM showed increased expression of proinflammatory genes and increased activity of profibrotic transcription factors relative to LPS-derived cells, while LPS-derived *Gpnmb* RecAM showed greater evidence of homeostatic and antiinflammatory gene expression and transcription factor activity.

## Discussion

Macrophages play critical roles in tissue repair and fibrosis. However, to our knowledge, no studies have comprehensively evaluated transcriptional profiles of macrophage subsets in nonfibrotic versus fibrotic models of tissue repair. To achieve this, we used scRNA-Seq to evaluate lineage-traced and compartment-labeled lung macrophages during homeostasis as well as over the time course of injury and repair following LPS and bleomycin challenge. Counter to our initial hypothesis, we identified no model-specific subsets. Instead, macrophage subset identities were most robustly defined by anatomic location and ontogeny and were conserved across lung injury models. Importantly, this included a subset of putatively “profibrotic” recruited macrophages marked by *Gpnmb*, *Spp1*, and other “fibrotic” genes. Collectively, our data support that “profibrotic” and “prorepair” macrophages represent the same subset that arises from monocytes in response to a variety of injury mechanisms. Thus, the presence of *Gpnmb* RecAM does not portend a specific repair outcome. Rather, time-dependent shifts in macrophage programming and persistence after injury are associated with the development of fibrosis. Accordingly, we suggest that recruitment of *Gpnmb*-expressing macrophages represents a conserved response to injury that is common to all forms of tissue repair.

Macrophages with transcriptional profiles similar to the *Gpnmb* RecAM lung macrophages identified in our study have been commonly implicated as “profibrotic” macrophages in the lung, liver, heart, and kidney (49, 63–65). However, as we demonstrate, this subset is clearly present in nonfibrotic lung disease. Moreover, macrophages with similar gene signatures have been described in a number of nonfibrotic diseases. For example, conserved *Gpnmb* RecAM genes (e.g., *Cd63*, *Fabp5*, *Lgals3*, and *Gpnmb*) are hallmarks of lipid-associated macrophages found in the tumor microenvironment and chronically inflamed liver and adipose tissue (66–68). Similarly, *Gpnmb*, *Trem2*, *Spp1*, and *CD9* distinguish disease-associated foamy macrophages from aortic intimal resident macrophages in atherosclerotic plaques from humans and mice, whereas *Gpnmb*, *Fam20c*, and growth factor–related genes enriched in *Gpnmb* RecAM (e.g., *Gdf15*, *Igf1*, and *Igf2r*) are hallmarks of a recently described macrophage subset essential for muscle repair (69). This is in agreement with our analysis showing that healing skin wounds in mice contained a macrophage subset enriched for the *Gpnmb* RecAM gene signature. Taken as a whole, these findings suggest that, while macrophages with a *Gpnmb* RecAM signature may be present during fibrosis, they are not sufficient to cause fibrotic tissue remodeling. Rather, we suggest that *Gpnmb*-recruited macrophages arise as part of a conserved response program to tissue injury across organs and species.

We also identified small numbers of *Gpnmb* RecAM present in the lungs of naive mice. While we cannot rule out the possibility that the presence of these cells is an artifact of lung digestion and tissue processing, we note that a similar subset of AMs has been documented in BAL form healthy humans (51). We therefore postulate that the presence of these cells in the naive lung represents a constitutive homeostatic tissue repair program. This may reflect continuous low-level alveolar damage as a consequence of inhaled exposures or constitutive cell turnover as part of a general tissue maintenance program.

Our data demonstrate that transcriptional profiles of RecAM and RecIM subsets from bleomycin- versus LPS-treated mice are nearly identical in the early phase of injury. This suggests a conserved program of monocyte differentiation. Indeed, a paradigm of conservation of monocyte differentiation programs across tissues and injury models was recently proposed (70). Sanin et al. used scRNA-Seq to compare monocyte-derived macrophages from mice infected with bacteria or helminths in adipose, vascular, and stromal tissue. Their analysis demonstrated 4 core monocyte programming signatures regardless of tissue or infection model, which were further validated in scRNA-Seq datasets of nerve, tumor, liver, lung, heart, retina, and skeletal muscle pathology. Our data do not discount the possibility that monocyte heterogeneity contributes to the injury response in LPS versus bleomycin. However, the overwhelming similarity of transcriptional profiles between recruited macrophage subsets at early time points in the bleomycin versus LPS models suggests that either that recruited macrophage subsets arise from the same precursors in each model or that, regardless of monocyte source, the pattern of differentiation is highly conserved.

The overall objective of our study was to identify features of macrophages that distinguish nonfibrotic repair from dysregulated repair with fibrosis. While we did not find a fibrosis-specific macrophage subset, we identified 2 key differences between nonfibrotic and fibrotic repair: (a) lack of homeostatic programming in *Gpnmb* RecAM in bleomycin relative to LPS and (b) persistence of recruited macrophage subsets. In contrast to early conservation of *Gpnmb* RecAM programming between LPS and bleomycin injury, we observed divergence at later time points in DEGs within this subset. *Gpnmb* RecAM in the LPS model became more RAM-like, while in the bleomycin model, they retained proinflammatory programming. It is notable that the divergent transcriptional programming had almost no relation to putative “profibrotic” marker genes, except for *Spp1* and *Emp1*. This further supports the concept that the “fibrotic” signature is predominantly linked to the injury-response identity of *Gpnmb* RecAM rather than a profibrotic function.

Differences in macrophage population sizes were apparent at all injury time points between models. In particular, absolute numbers of all clusters that contained recruited macrophages (including *Gpnmb* RecAM) remained elevated in bleomycin compared with LPS at day 14/15. Persistence of recruited macrophages may be a key feature of delayed resolution and fibrotic repair. Forced persistence of RecAM by caspase-8 inhibition resulted in prolonged albumin leak into BAL following LPS-induced lung injury in mice (71). Reciprocally, induced apoptosis during fibrotic repair of all IM and RecAM, all AM, or RecAM attenuated fibrosis (18, 19, 21). While factors including FasL and TNF-α have been shown to mediate RecAM apoptosis, the mechanisms by which *Gpnmb* RecAM persist in bleomycin relative to LPS are unknown. Additionally, the significance of persistence of the recruited IM subsets in bleomycin is unknown.

Overall, determining the signals that drive the divergence in *Gpnmb* RecAM programming may yield more disease-specific targets and allow for a more nuanced understanding of repair responses. Moreover, establishing the mechanisms by which the *Gpnmb* RecAM subset persists in the lungs in fibrotic injury may provide additional avenues for intervention. It is likely that crosstalk with other cell types including fibroblasts and epithelial cells in the lung microenvironment influence the persistence and programming of this macrophage subset. Understanding the persistence of the *Gpnmb* RecAM macrophage subset and its crosstalk with structural cells are key areas for future investigation.

Our study has several limitations. First, since our cell sorting strategy prioritized macrophage enrichment, we were underpowered to evaluate roles played by other cell types in driving fibrotic versus nonfibrotic tissue repair. Therefore, whether tissue repair outcomes are dictated by injury responses from a single-cell lineage versus multicellular networks remains an open question. Second, since our goal was to determine factors that lead to fibrosis rather than its resolution, we did not evaluate later time points following bleomycin injury. It therefore remains possible that additional macrophage subsets with unique programming appear at later time points in the bleomycin model and that these could present specific targets for intervention.

A central aspect of our study design incorporated genetic labeling of RIM prior to the onset of injury with compartment labeling of macrophages at the time of harvest. Since genetically labeled RIM were never detected in the luminal compartment as marked by lavage or IT CD45 staining, we conclude that RIM do not migrate to the airspaces. In comparison, we note that some cells in our *Gpnmb* RecAM cluster derived from IM sorts and that some cells in the *Irf7* RecIM cluster derived from the AM sorts. While these findings could reflect technical limitations in compartment sorting, we believe they are more closely in agreement with reports that RecIM serve as an obligatory intermediate between circulating monocytes and RecAM (72). Carefully timed lineage-tracing studies would be required to fully assess macrophage migration kinetics and developmental trajectories.

Lastly, while we focused primarily on interrogating *Gpnmb* RecAM and their role in the injury response, all 7 macrophage subsets exhibited differences in cell numbers and gene expression at various time points between the 2 injury models. Intriguingly, *Ear1^hi^* RAM from LPS versus bleomycin had differential gene expression that preceded differences in recruited macrophage subsets and persisted longer, possibly reflecting early and sustained outcome–associated shifts in RAM programming. Further analysis of other macrophage subsets is ongoing and may yield additional clues into the pathophysiology of fibrotic versus nonfibrotic repair.

To our knowledge, our study is the first to directly compare dynamic transcriptional profiles of macrophage subsets during fibrotic versus nonfibrotic lung tissue repair. Taken as a whole, our data show that anatomic compartment and cell origin are the main drivers of macrophage identity and that time- and model-dependent factors play much lesser roles. Our data further show that transcriptionally defined macrophage subsets — including a subset marked by *Gpnmb*, *Spp1*, and other “profibrotic” markers that was present in multiple human diseases and in health — were conserved across models. Accordingly, while this subset appears necessary for the development of fibrosis, its presence is not sufficient. Overall, our findings support a broader paradigm wherein recruited macrophages express a conserved injury response program following loss of tissue homeostasis. Their progression to tissue resident macrophage–like programming and numerical decline support rapid resolution of injury, while failure of these 2 elements contributes to persistent inflammation and pathologic fibrotic repair.

## Methods

Supplemental Methods are available online with this article.

### Sex as a biological variable.

Sex has been previously shown to affect both lung inflammation and development of fibrosis in murine LPS and bleomycin models (73, 74). To minimize variability between experiments and to enhance statistical power, only male mice were used in scRNA-Seq experiments in this study.

### Mice.

All mice used were *Cx3cr1^ERT2-Cre^* × *R26^Stop(fl/fl)tdTomato^* (referred to as Cx3cr1-TdTomato) reporter mice. These were generated in our facility at National Jewish Health via breeding of male *Cx3cr1^ERT2-Cre^* mice and female *R26^Stop(fl/fl)tdTomato^* mice purchased from The Jackson Laboratory (38). Male mice between 8 and 10 weeks of age were used for all experiments. We developed a macrophage sorting and sequencing strategy to preserve tissue compartment information (AM versus IM) and IM origin (RIM versus RecIM). Differentiating the airspace versus interstitial tissue compartment was achieved by i.t. instillation of anti-CD45 antibody (clone 30-F11, BD Biosciences) after euthanasia and prior to lung harvesting. Thus, all i.t. CD45^+^ labeled cells were assigned to the airspace compartment during flow cytometry sorting. To distinguish IM origin, we used an established RIM lineage tracing model (Cx3cr1-TdTomato pulse-wait) to label RIM prior to lung injury (13, 38). *Cx3cr1* is expressed by all RIM as well as monocytes and dendritic cells. A pulse of tamoxifen labeled all of these cell types; however, after a 4-week wait period, only the RIM remained labeled. Thus, TdTomato marks RIM in our study.

### Mouse treatments.

Mice received 3 doses of i.p. tamoxifen (Sigma Pharmaceuticals) over one week. Tamoxifen was suspended in corn oil and delivered at a concentration of 0.2 mg/g per dose. Animals were rested for 4 weeks after the final tamoxifen dose (13, 38). *E*. *coli* LPS O55:B5 (List Biological Laboratories) was instilled into the tracheas of mice sedated with isoflurane (Baxter) at a concentration of 20 μg in 50 μL of PBS using a modified gel-loading tip with direct laryngoscopy (13). Bleomycin (TEVA Pharmaceuticals) was administered at a dose of 1.5 U/kg in 50 μL of PBS by the same method (21). Mice were euthanized by i.p. injection of 10% Fatal Plus solution (Vortech Pharmaceuticals). Three to 4 mice were treated per time point in each inflammatory model.

### Macrophage isolation and sequencing.

After euthanasia, an 18-gauge catheter was inserted into the trachea, and airspace immune cells were labeled by i.t. instillation of 800 μL anti-CD45 antibody diluted 1:200 in PBS. Four minutes later, bronchoalveolar lavage (BAL) was performed to remove excess unbound anti-CD45 antibody from the airspaces (13). BAL cells were added to minced lung tissue, and samples were digested in 0.4 mg/mL Liberase TM (Roche) as previously described (75). Macrophage subsets were sorted from digested lung tissue as previously described and as shown in Supplemental Figure 2 (13). Briefly, macrophages were gated based on expression of CD64 (clone X54-5/7.1, BioLegend) and CD88 (clone 20/70, BioLegend), and AMs were separated from IMs based on i.t. anti-CD45 staining. Within the IM compartment, RIM and RecIM were distinguished by expression of the Cx3cr1-TdTomato reporter. Addition of a separate anti-CD45 antibody with a different fluorochrome to the staining panel allowed for sorting of the total CD45^+^ population to normalize across samples and analyze cell cluster proportions. At least 20,000 cells were sorted for each subset (RAM, RIM, RecIM, total CD45) on a Sony ICyte Synergy cell sorter. Cell counts were confirmed by hemocytometer, and samples were resuspended at 700–1,200 cells/μL in 0.06% BSA for single-cell capture. We aimed to capture 3,000 cells from each macrophage sort and 5,000 cells from each total CD45^+^ sort.

### Quality control and clustering.

Transcripts per cell were counted for each library using CellRanger, and cells with high mitochondrial transcript counts (>15%), low total counts (<1,000), or low numbers of expressed genes (<500) were excluded (76, 77). Remaining cells were integrated using a standard Seurat workflow with the reciprocal principal component analysis method and clustered using the standard Seurat Louvain method (78). Cluster cell types were manually annotated based on differentially expressed marker genes cross-referenced with murine LungMAP data (79). To identify macrophage subsets, we subclustered cells with macrophage identities. The number of clusters was set based on the biological relevance of genes separating each cluster.

### Statistics.

Differential gene expression analyses were performed using pseudo-bulk methods (80, 81). Pathway and gene set enrichment analyses were performed using the enrichR R packages with the Reactome 2022, KEGG 2019 mouse, GO Biological Process 2021, GO Molecular Function 2018, and GO Cellular Component 2018 databases (82). Pathway network visualization was performed with the enrichment map feature in the Cytoscape platform (41, 42, 83). Testing for enrichment of the profibrotic gene set and the cluster-specific *Gpnmb* RecAM gene set was performed by hypergeometric tests in the hypeR package (20, 84). Gene lists for human IPF macrophages, healthy human macrophages, human COVID-19 macrophages, human asthma macrophages, mouse pneumocystis macrophages, and mouse skin wound macrophages were acquired from published datasets (24, 29, 52, 53, 54, 85). Transcription factor analysis was performed using the decoupleR univariate linear model method and the CollecTRI database of gene regulatory networks and transcription factors (86, 87).

### Study approval.

This study was approved by and performed in accordance with IACUC protocols at National Jewish Health.

### Data availability.

Raw sequence files and processed files are available on the Gene Expression Omnibus (accession no. GSE280003). Values for all data points in graphs are reported in the Supporting Data Values file.

## Author contributions

EMK, EFR, ALM, and WJJ designed the research studies. EMK, SAM, and ALM performed experiments. YZ, CMM, BS, KCA, and BV performed statistical analyses. EMK, YZ, CMM, PKM, CME, KJM, EFR, ALM, and WJJ analyzed and interpreted the data as well as wrote, reviewed, and revised the manuscript.

## Supplementary Material

Supplemental data

Supplemental table 1

Supplemental table 2

Supplemental table 3

Supplemental table 4

Supplemental table 5

Supplemental table 6

Supplemental table 7

Supplemental table 8

Supporting data values

## Version 1 11/07/2024

In-Press Preview

## Version 2 12/20/2024

Electronic publication
