# Supplementary material for: Gpnmb and Spp1 mark a conserved macrophage injury response masking fibrosis-specific programming in the lung
Source: JCI Insight. 2024 Dec 20;9(24):e182700. doi: 10.1172/jci.insight.182700 (PMC11665561; doi:10.1172/jci.insight.182700)
Supplement: Supplemental data [file jciinsight-9-182700-s175.pdf]

## Statistical Methods

### Bioinformatics Processing and Quality Control for scRNAseq

Demultiplexing, alignment to the mm10-3.0.0 transcriptome, and UMI-collapsing was performed using the Cellranger toolkit (v3.1.0, 10X Genomics, Pleasanton, CA) (1). Data were collected on 297,472 cells from 21 mice. All post-quantification quality control, normalization, integration, clustering, and marker finding were performed in Seurat (v4.0.0, Satija Lab) (2). We filtered out low quality cells with less than 500 genes detected or with greater than 15% of mapped reads originating from the mitochondrial genome. We also removed cells with total UMI counts less than 1,000. This caused the Bleomycin Day 7 IMRes sort from replicate 1 to be excluded entirely. The quality-controlled dataset consisted of 242,064 cells.

### Integration and Preliminary Clustering

UMI counts were log-normalized and data from all samples and sorts were combined using single cell integration implemented in Seurat, which identifies mutual nearest neighbor (MNN) cells across pairwise subjects to use as “anchors” to perform batch correction. The top 2,000 were selected based on the “vst” method of FindVariableFeatures for each sample and reciprocal principal component analysis was used to integrate all samples, using 3,000 features, two cell anchors (k.anchor = 1, and the top 30 dimensions of the underlying data (dims = 1:30). For visualization, we reduced variation to two dimensions using Uniform Manifold Approximation and Projection (UMAP; n.neighbors = 30, min.dist = 0.3). Integrated data were clustered using the standard Seurat Louvain method (dims = 1:50) with a resolution parameter of 0.5. This algorithm identified 34 clusters. To identify cluster markers, we carried out pairwise differential expression analysis comparing log-normalized expression in each cluster to all others using a Wilcoxon rank sum test. Preliminary cell type markers were identified as genes exhibiting significant upregulation when compared against all other clusters, defined by having a Bonferroni adjusted p-value < 0.05, a log fold change > 0.4. Cell types were annotated by comparing these results against mouse LungMAP marker genes (3).1 sample from the Bleo Day 3 group was removed from downstream analysis as the mouse erroneously did not receive bleomycin treatment (226,742 cells remaining).

### Macrophage Sub-Clustering and Subset Markers

To identify macrophage subsets, clusters 0, 1, 3, 6, 10, 12, and 15 (N=105,927 cells) were sub-clustered as described above using 25 PCs, a resolution parameter of 0.3 and the SLM algorithm, resulting in 10 sub-clusters, including a proliferating cluster (preliminary sub-cluster 8). The proliferative sub-cluster and cells originating from the fibroblast sort were removed and a final macrophage sub-clustering was performed on the remaining N=99,589 cells, again, using 25 PCs and a resolution parameter of 0.3. Macrophage subset markers were identified using Seurat’s FindConservedMarkers function. We required conserved markers to have significant upregulation (average log fold change > 0.5, expression in >10% of cells, and a Bonferroni adjusted p-value < 0.05) in at least 80% of the samples with at least 25 cells in the subset. Macrophage sub-cluster 3 (N=11,366 cells) did not have any distinct markers and was largely distinguished by

having cells with low total UMI counts. As this appeared to be a cluster of low-quality cells, it was removed from downstream analyses.

**Differential Gene Expression Between Inflammation Model and Timepoints**

Differentially expressed genes between inflammation models and time points within each macrophage subset were found using pseudo-bulk methods to account for clustering of cells within samples (4, 5). Briefly, a pseudobulk representation was constructed by summing over counts for all cells in a particular group and replicate for a macrophage subset. We performed differential expression analyses on genes with pseudobulk counts >10 in at least 3 samples (N=12,948 genes), using negative binomial regression models implemented in edgeR (6). The following conditions were included as predictors in the model: homeostasis, Bleo Day 3, Bleo Day 7, Bleo Day 14, LPS Day 3, LPS Day 6, and LPS day 15. We used likelihood ratio tests to test for changes over time within each inflammation model and performed contrasts to test for differences between timepoints within each inflammation model and for differences between Bleo and LPS at each time point (Bleo Day 3 vs LPS Day 3; Bleo Day 7 vs LPS Day 6; Bleo Day 14 vs LPS Day 15). A Benjamini-Hochberg adjusted p-value threshold of 0.05 was used to determine statistical significance.

**Pathway and Gene Set Enrichment Analyses**

Pathway enrichment analyses were performed on marker lists and lists of differentially expressed genes using the enrichR R packages with the Reactome 2022, KEGG 2019 Mouse, GO Biological Process 2021, GO Molecular Function 2018, and GO Cellular Component_2018 databases (7). In addition, we performed custom enrichment analyses using hypergeometric tests implemented in the hypeR package to test for enrichment of the pro-fibrotic gene set reported by Joshi *et al.* in the marker lists of each macrophage subset (8, 9).

We also used the hypeR package to test for enrichment of the same profibrotic gene set and markers from our *Gpnmb* RecAM cluster in several publicly available human and mouse scRNA-seq datasets. Marker genes for macrophage subsets from healthy human lung tissue were published by Morse *et al.* (10). IPF lung tissue macrophage marker genes were published by Reyfman *et al.* (11). Human COVID-19 macrophages were sequenced from bronchoalveolar lavage and marker genes were published by Grant *et al.* (12). The dataset for macrophages in human asthma was generated from endobronchial brushings and published by Alladina *et al.* (13). Sequencing data for macrophages from lung tissue of mice infected with *Pneumocystis murina* were published by Wang *et al.* and sequencing of macrophages from skin wounds was published by Ma *et al.* (14, 15).

**Gene Scores**

Pro-fibrotic gene scores were calculated for each cell in the macrophage clustering by taking the average of the centered and scaled expression of genes in the pro-fibrotic gene set. Expression scores were compared between timepoints within the *Gpnmb* RecAM cluster using linear mixed models with a random effect for replicate to account for

clustering of cells within a sample. We also compared scores between clusters within the homeostasis condition using similar models.

### Transcription Factor Analysis

DecoupleR was used to calculate transcription factor activity scores for all cells using the Univariate Linear Model (ULM) method and CollecTRI, a curated meta-resource database of gene regulatory networks and transcription factors (16, 17). We compared activity scores between macrophage subsets, using linear mixed models with a random effect for replicate to account for clustering of cells within samples.

### Cytoscape

The lists of up-regulated genes were each input individually into the BiNGO Cytoscape plugin to assess overrepresentation of gene ontology categories (18, 19). Specifically, we used the hypergeometric test with an FDR threshold of 0.05 with the GO Biological Processes ontology for Mus musculus while using the whole annotation as a reference set. The resulting networks of significant ontology terms were then visualized using the Enrichment Map Cytoscape plugin (20).


1. Zheng GXY, Terry JM, Belgrader P, Ryvkin P, Bent ZW, Wilson R, et al. Massively parallel digital transcriptional profiling of single cells. *Nat Commun.* 2017;8(1):14049.
2. Hao Y, Hao S, Andersen-Nissen E, Mauck WM, 3rd, Zheng S, Butler A, et al. Integrated analysis of multimodal single-cell data. *Cell.* 2021;184(13):3573-87.e29.
3. Guo M, Morley MP, Jiang C, Wu Y, Li G, Du Y, et al. Guided construction of single cell reference for human and mouse lung. *Nat Commun.* 2023;14(1):4566.
4. Helena LC, Charlotte S, Pierre-Luc G, Daniela C, Ludovic C, Catarina R, et al. On the discovery of subpopulation-specific state transitions from multi-sample multi-condition single-cell RNA sequencing data. *bioRxiv.* 2020:713412.
5. Lun ATL, and Marioni JC. Overcoming confounding plate effects in differential expression analyses of single-cell RNA-seq data. *Biostatistics.* 2017;18(3):451-64.
6. Robinson MD, McCarthy DJ, and Smyth GK. edgeR: a Bioconductor package for differential expression analysis of digital gene expression data. *Bioinformatics.* 2010;26(1):139-40.
7. Kuleshov MV, Jones MR, Rouillard AD, Fernandez NF, Duan Q, Wang Z, et al. Enrichr: a comprehensive gene set enrichment analysis web server 2016 update. *Nucleic Acids Res.* 2016;44(W1):W90-7.
8. Federico A, and Monti S. hypeR: an R package for geneset enrichment workflows. *Bioinformatics.* 2020;36(4):1307-8.
9. Joshi N, Watanabe S, Verma R, Jablonski RP, Chen CI, Cheresh P, et al. A spatially restricted fibrotic niche in pulmonary fibrosis is sustained by M-CSF/M-CSFR signalling in monocyte-derived alveolar macrophages. *Eur Respir J.* 2020;55(1).
10. Morse C, Tabib T, Sembrat J, Buschur KL, Bittar HT, Valenzi E, et al. Proliferating SPP1/MERTK-expressing macrophages in idiopathic pulmonary fibrosis. *Eur Respir J.* 2019;54(2).
11. Reyfman PA, Walter JM, Joshi N, Anekalla KR, McQuattie-Pimentel AC, Chiu S, et al. Single-Cell Transcriptomic Analysis of Human Lung Provides Insights into the Pathobiology of Pulmonary Fibrosis. *Am J Respir Crit Care Med.* 2019;199(12):1517-36.

12. Grant RA, Morales-Nebreda L, Markov NS, Swaminathan S, Querrey M, Guzman ER, et al. Circuits between infected macrophages and T cells in SARS-CoV-2 pneumonia. *Nature.* 2021;590(7847):635-41.
13. Alladina J, Smith NP, Kooistra T, Slowikowski K, Kernin IJ, Deguine J, et al. A human model of asthma exacerbation reveals transcriptional programs and cell circuits specific to allergic asthma. *Sci Immunol.* 2023;8(83):eabq6352.
14. Wang Y, Li K, Zhao W, Liu Y, Li T, Yang HQ, et al. Integrated multi-omics analyses reveal the altered transcriptomic characteristics of pulmonary macrophages in immunocompromised hosts with Pneumocystis pneumonia. *Front Immunol.* 2023;14:1179094.
15. Ma J, Song R, Liu C, Cao G, Zhang G, Wu Z, et al. Single-cell RNA-Seq analysis of diabetic wound macrophages in STZ-induced mice. *J Cell Commun Signal.* 2023;17(1):103-20.
16. Badia IMP, Vélez Santiago J, Braunger J, Geiss C, Dimitrov D, Müller-Dott S, et al. decoupleR: ensemble of computational methods to infer biological activities from omics data. *Bioinform Adv.* 2022;2(1):vbac016.
17. Müller-Dott S, Tsirvouli E, Vazquez M, Ramirez Flores RO, Badia IMP, Fallegger R, et al. Expanding the coverage of regulons from high-confidence prior knowledge for accurate estimation of transcription factor activities. *Nucleic Acids Res.* 2023;51(20):10934-49.
18. Maere S, Heymans K, and Kuiper M. BiNGO: a Cytoscape plugin to assess overrepresentation of gene ontology categories in biological networks. *Bioinformatics.* 2005;21(16):3448-9.
19. Shannon P, Markiel A, Ozier O, Baliga NS, Wang JT, Ramage D, et al. Cytoscape: A Software Environment for Integrated Models of Biomolecular Interaction Networks. *Genome Research.* 2003;13(11):2498-504.
20. Merico D, Isserlin R, Stueker O, Emili A, and Bader GD. Enrichment map: a network-based method for gene-set enrichment visualization and interpretation. *PLoS One.* 2010;5(11):e13984.

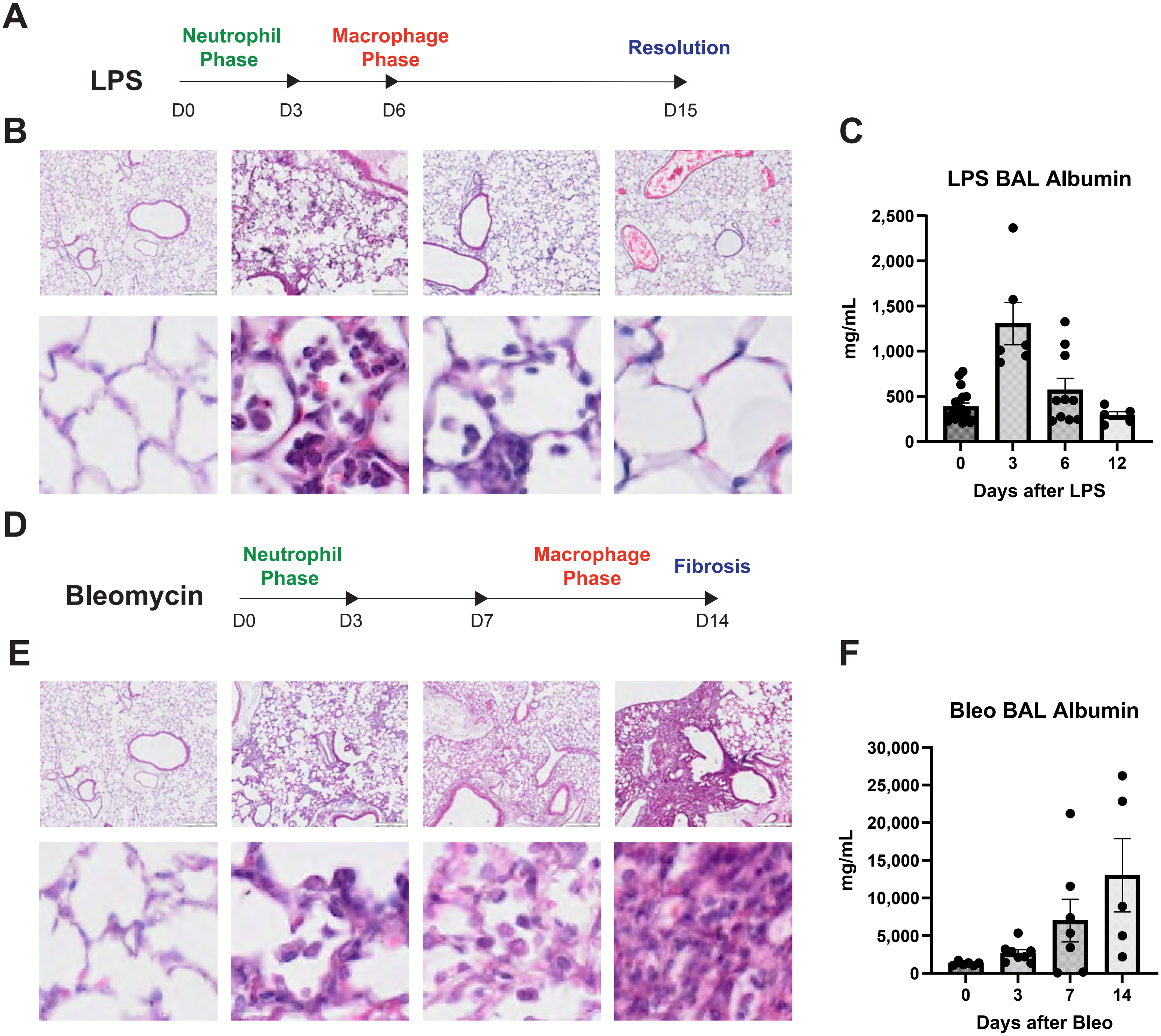


**Supplementary Figure 1. I.t. LPS causes resolving lung injury, while i.t. bleomycin causes prolonged injury with fibrosis.** A) Intratracheal LPS is characterized by early infiltration of neutrophils followed by the peak of macrophage numbers and resolution of injury and inflammation by 15 days. B) Hematoxylin and eosin staining of murine lung tissue sections at the corresponding time points. C) Bronchoalveolar lavage (BAL) albumin concentrations at days 0, 3, 6, and 12 after LPS. D) Intratracheal bleomycin is characterized by a much smaller degree of early neutrophil infiltration followed by expanding macrophage numbers and fibrosis detectable by 14 days. E) Hematoxylin and eosin staining of murine lung tissue from days 0, 3, 7, and 14 after bleomycin. F) BAL albumin concentrations at 0, 3, 7, and 14 days after bleomycin.

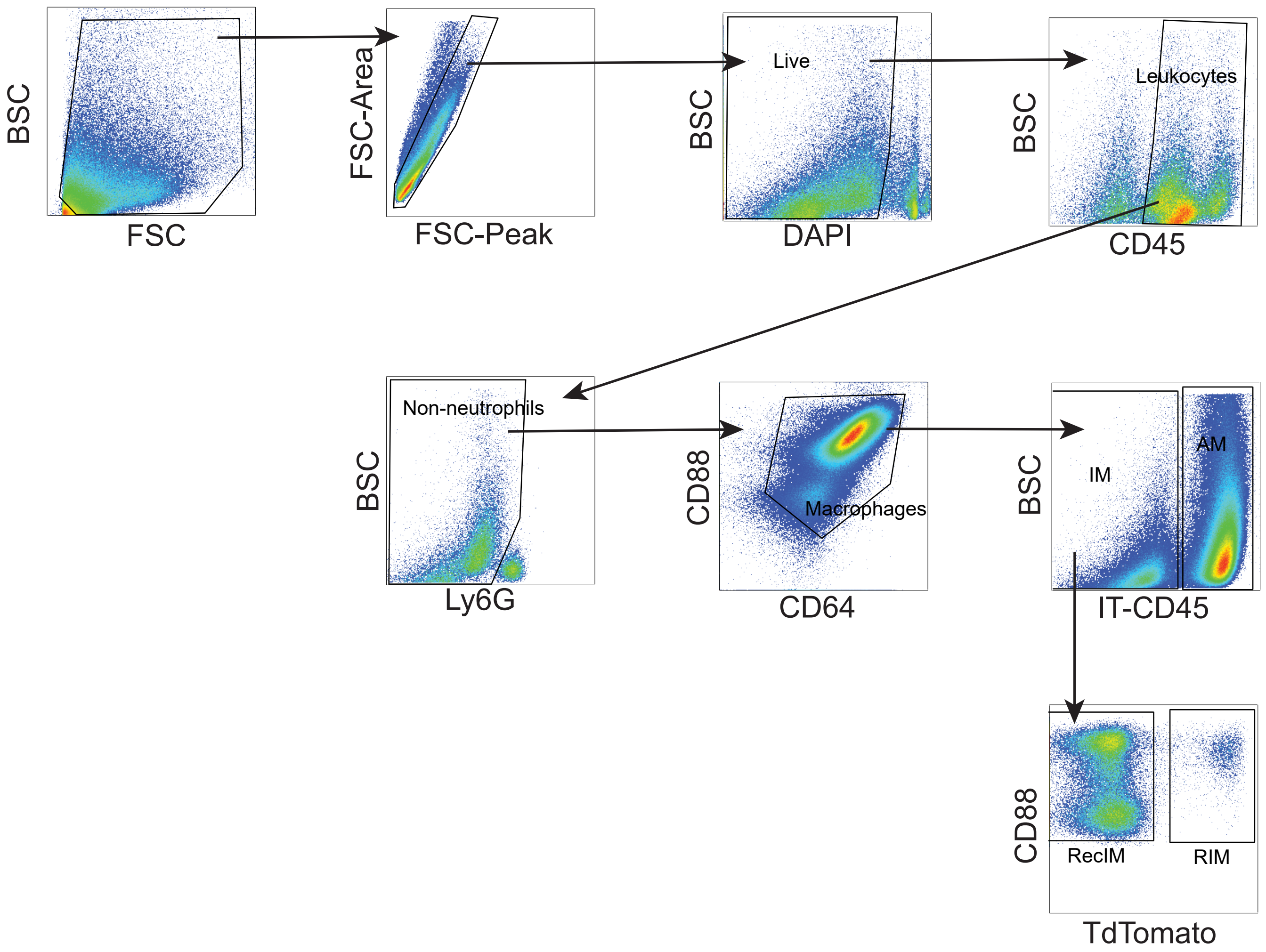


**Supplementary Figure 2. Gating strategy for sorting macrophage subsets for sequencing.** Representative gating of an LPS day 6 lung sample. Doublets were excluded, followed by exclusion of dead cells with DAPI, and CD45 was used to gate all leukocytes. Neutrophils were excluded with Ly6G, and macrophages were identified as CD88+ CD64+ cells. Intratracheal (IT) CD45+ macrophages were sorted and captured for sequencing. Interstitial macrophages (IMs) were identified by absence of IT CD45 staining. Resident IM (RIM) versus recruited IMs (RecIM) were distinguished using TdTomato. These two subsets were sorted and sequenced separately. A sample of the total CD45 cells was also sorted and sequenced (not shown).

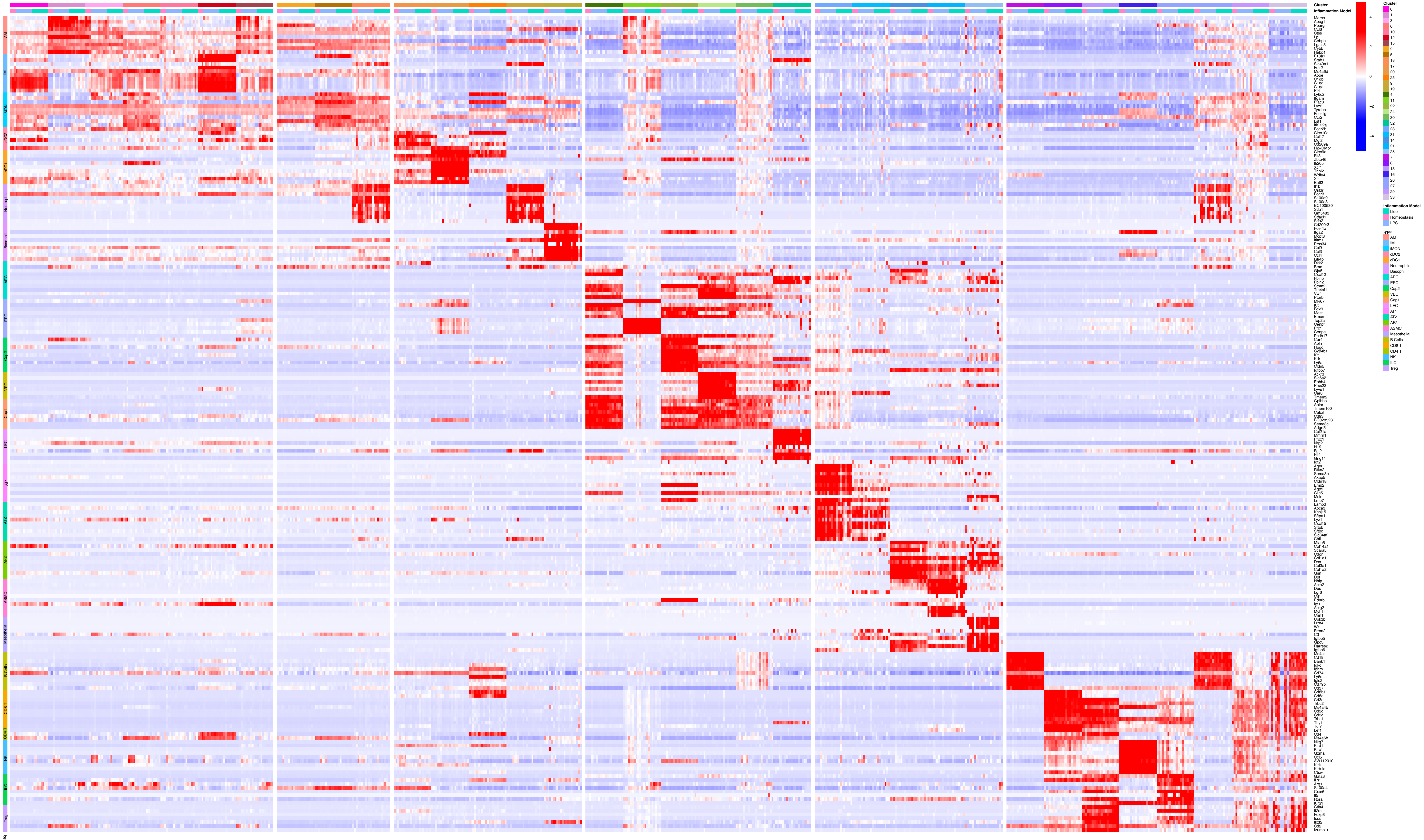

**Supplementary Figure 3. Heatmap of LungMAP cell identity genes corresponding to the 34 Seurat clusters.** Relative expression of murine LungMAP marker genes defining 25 different cell types in the 34 Seurat clusters. The 34 clusters are shown in columns with each mouse replicate from the 3 inflammation models shown. Genes corresponding to 25 LungMAP cell types are shown in rows, and gene expression is scaled across rows.

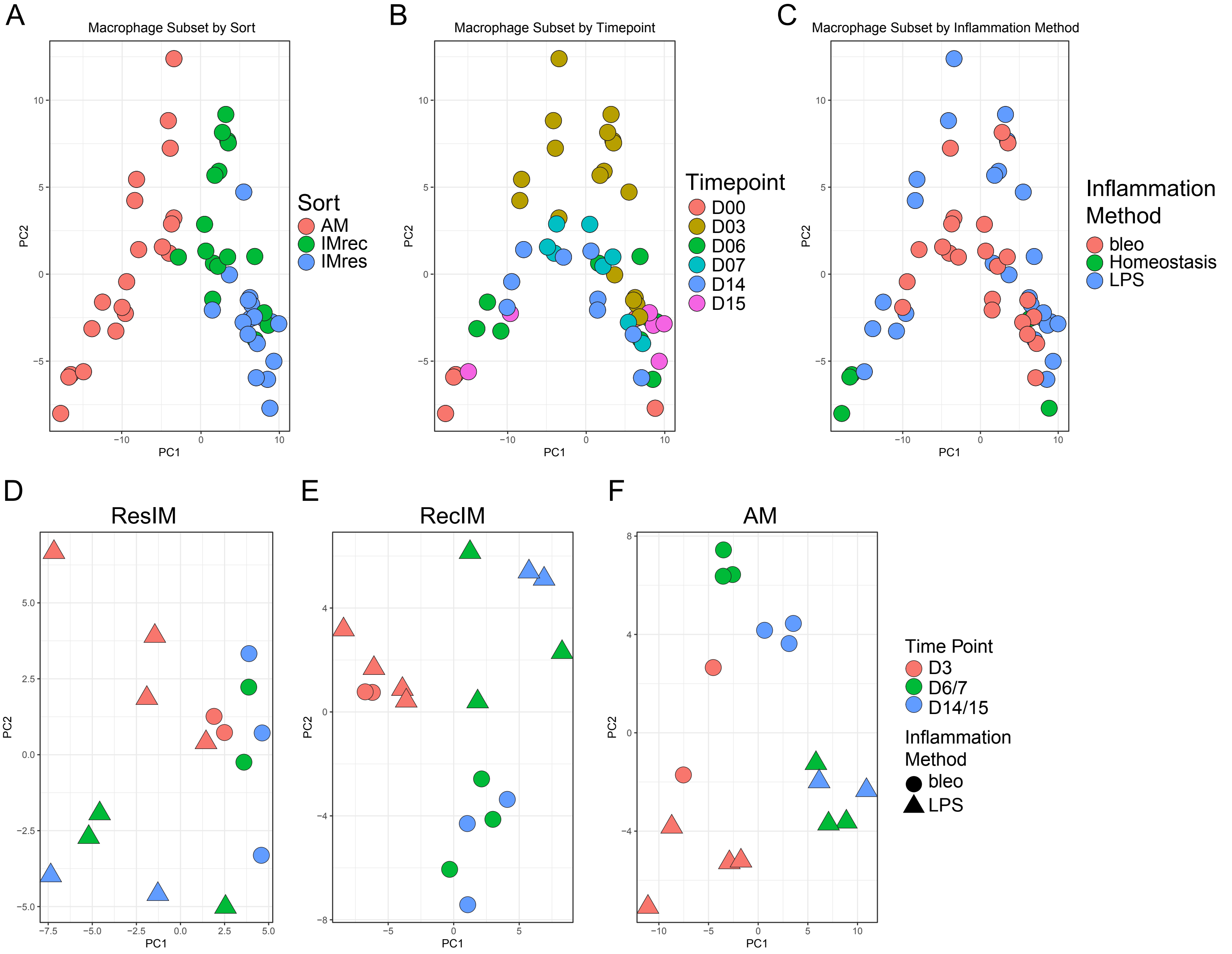


**Supplementary Figure 4. Macrophage origin and compartment are stronger drivers of gene expression than injury model or time point.** Principal component analysis was performed on the average log-normalized expression of macrophages from each sort (RIM, RecIM, AM) at each model time point. A) All sorts and time points are plotted, and data points are color-coded by sort. B) All sorts and time points plotted and data points are color-coded by time point. C) All sorts and time points are plotted, and data points are color-coded by inflammation method. D-F) RIM, RecIM, and AM sorts are plotted and color coded by time point. Injury model is indicated by triangles (LPS) or circles (bleomycin).

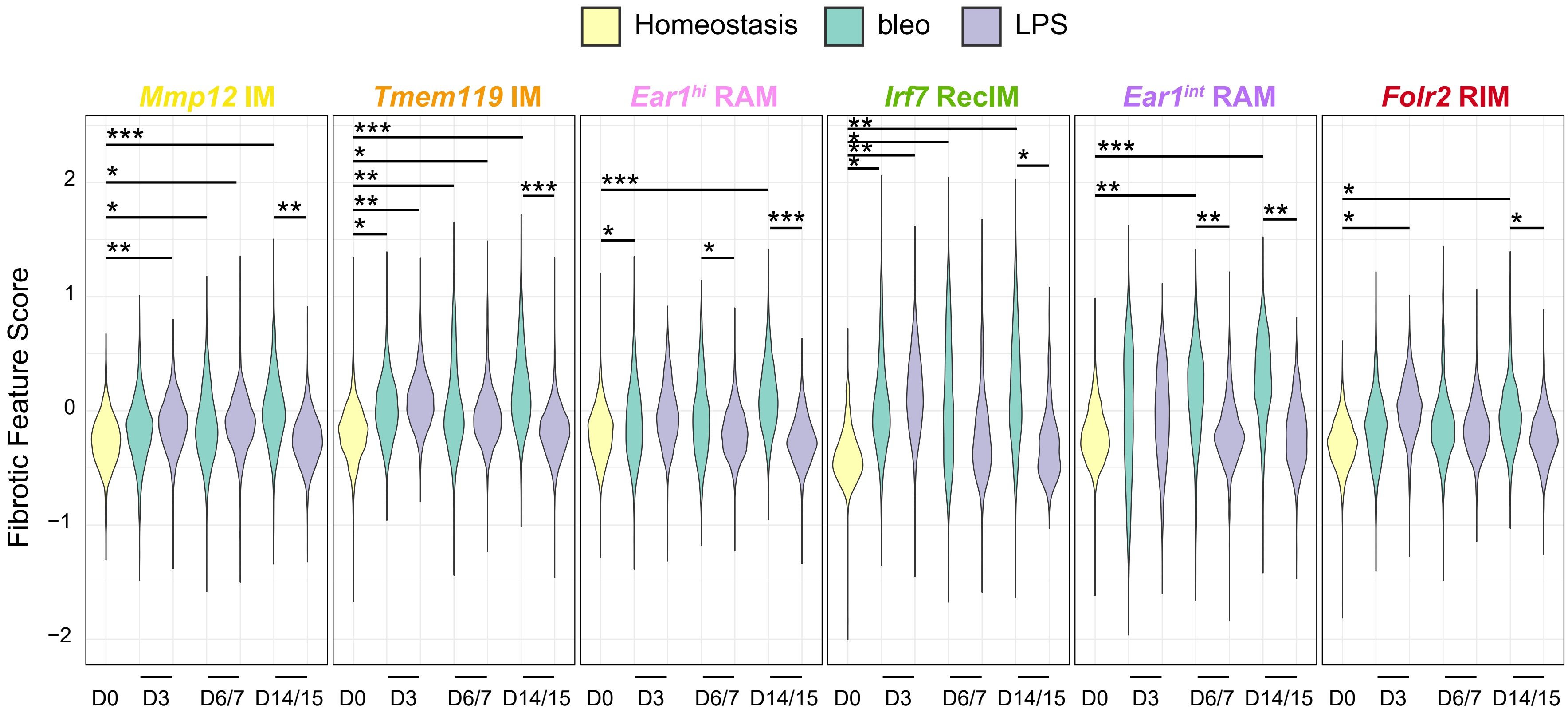


**Supplementary Figure 5. Fibrotic features scores in macrophage clusters over time.** Violin plots of fibrotic features scores calculated for each cluster (excluding Gpnmb RecAM, which are shown in figure 4b) for homeostasis and each time point in each model. (* p.adj. <0.05, ** p.adj. <0.005, *** p.adj. <0.0005, 1 random effect).
